# Artificial intelligence for the management of pancreatic diseases

**DOI:** 10.1111/den.13875

**Published:** 2020-12-05

**Authors:** Myrte Gorris, Sanne A. Hoogenboom, Michael B. Wallace, Jeanin E. van Hooft

**Affiliations:** ^1^ Department of Gastroenterology and Hepatology Amsterdam Gastroenterology Endocrinology Metabolism Amsterdam University Medical Centers University of Amsterdam Amsterdam The Netherlands; ^2^ Department of Gastroenterology and Hepatology Leiden University Medical Center Leiden The Netherlands; ^3^ Department of Gastroenterology and Hepatology Mayo Clinic Jacksonville Jacksonville USA

**Keywords:** artificial intelligence, diagnosis, computer‐assisted, diagnostic imaging, endoscopy, pancreatic diseases

## Abstract

Novel artificial intelligence techniques are emerging in all fields of healthcare, including gastroenterology. The aim of this review is to give an overview of artificial intelligence applications in the management of pancreatic diseases. We performed a systematic literature search in PubMed and Medline up to May 2020 to identify relevant articles. Our results showed that the development of machine‐learning based applications is rapidly evolving in the management of pancreatic diseases, guiding precision medicine in clinical, endoscopic and radiologic settings. Before implementation into clinical practice, further research should focus on the external validation of novel techniques, clarifying the accuracy and robustness of these models.

## INTRODUCTION

The artificial intelligence (AI) health market is growing explosively to a market size of $6.6 billion, with a compound annual growth rate of 40%.^1^ AI techniques are emerging, especially in imaging‐based specialties like radiology and gastroenterology. Modern imaging modalities, including endoscopy and cross‐sectional imaging, contain far more visual information than the human eye can distinguish. In addition, the digitalization of health records constituted an almost infinite storage of patient data. Several AI‐based methods have been employed to mine predictive patterns in this nearly endless source of data. In this review, we aim to give an overview of the current evidence on AI applications in pancreatic diseases, comprising clinical, endoscopic and radiologic applications. We performed a literature search for relevant articles on PubMed and Medline from January 2000 through May 2020 using keywords as *pancreas* and *machine learning* (Table S1).

## ARTIFICIAL INTELLIGENCE

**Figure 1:**
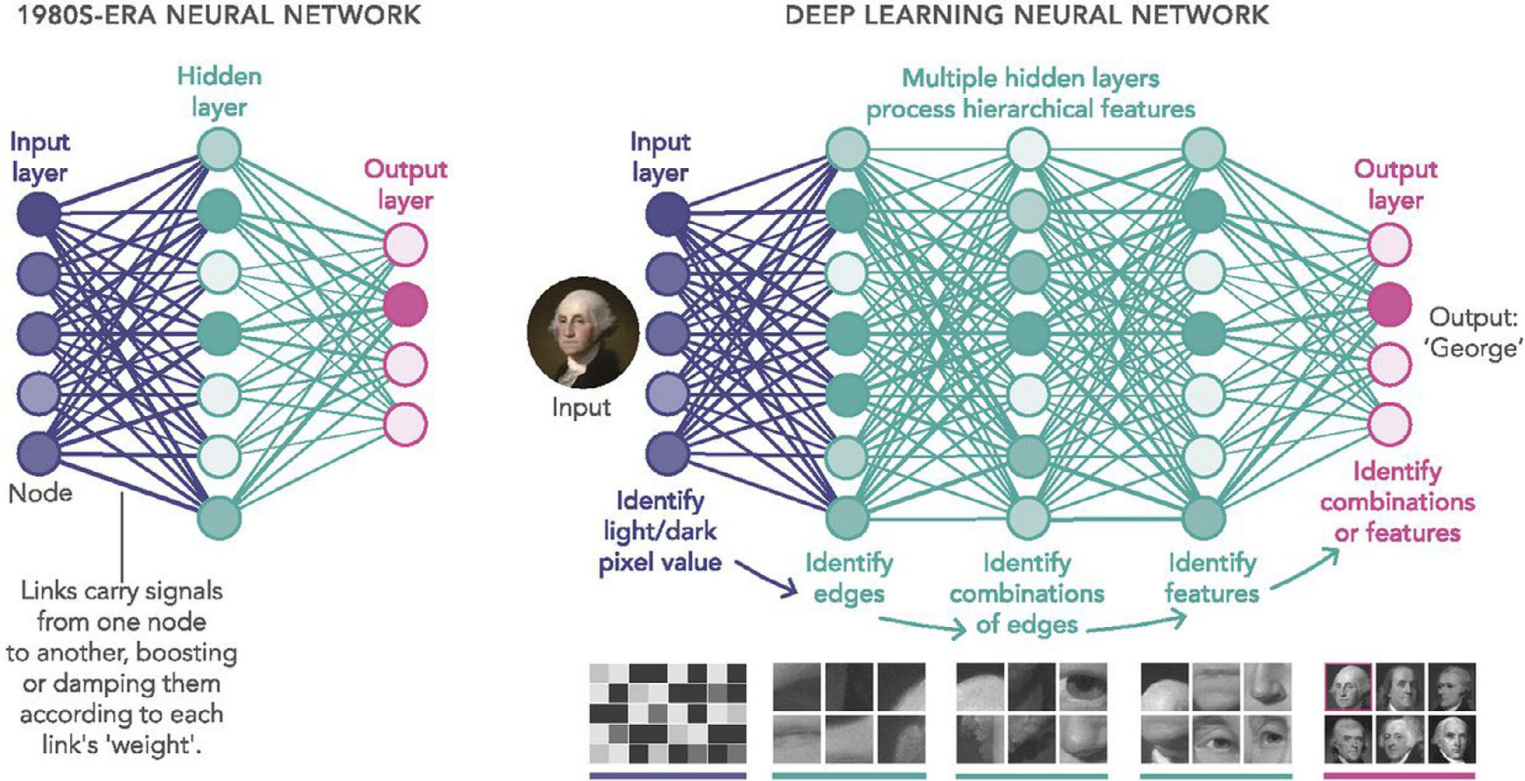
Neural networks. Neural networks send signals from the input layer through a network of nodes. The network is trained by the process of adjusting the weights that amplify or damp the transmitted signals, carried by the links between the nodes. Deep learning networks have dozens of hidden layers and can model complex relationships within data. Reprinted with permission from M. Mitchell Waldrop. News Feature: What are the limits of deep learning? *Proceedings of the National Academy of Sciences*. Jan 2019, 116 (4) 1074–1077. Created by Lucy Reading‐Ikkanda.

Artificial intelligence is an umbrella term for forms of *human* intelligence demonstrated by a computer, for example learning and problem‐solving.^2^ Machine learning (ML) is defined as the ability of a computer to learn and recognize patterns by analyzing data and improve their performance through experience.^3^ In traditional ML methods, like support vector machines (SVM) and random forests (RF), predefined features are necessary for accurate prediction. These conventional models are trained to predict the correct outcome based on predefined extracted features. In contrast, a subset of ML called deep learning (DL), does not require (manual) feature extraction. The architecture of DL algorithms is loosely inspired by interconnected neurons in the human brain and form a multilayered artificial neural network (ANN). The most commonly applied DL methods are convolutional neural networks (CNN), containing deep layers of filtering operations (convolutions) capable of modeling very complex relationships within data (Fig. 1).^4^ DL models utilize and analyze data to learn higher‐level features and derive an outcome based on these features.^5^ Although some DL models are outperforming humans in specific tasks, there are certain limitations that withhold broad application in clinical practice.^6^, ^7^ To start, a DL model can be excellent in predicting an outcome, but they do not explain upon which features the prediction is based (black‐box). Secondly, training a DL algorithm requires extensive well‐annotated datasets, which are of limited availability.^8^ The problem of data scarcity can be partly solved by two methods, namely data augmentation and transfer learning.^9^ Data augmentation is a technique in which the training dataset is artificially expanded by slightly altering the available images, such as flipping and rotating the images. Transfer learning is the process of pre‐training a model with a general image database like ImageNet, before training and fine‐tuning the model on a specific task.^10^ For example, an algorithm can be pre‐trained to recognize simple edges and shapes based on common objects which may later be transfer learned to the actual task. However, the true benefit of transfer learning for the analysis of medical images is under debate and needs to be further elucidated.^11^

### Artificial intelligence in the management of pancreatic diseases

In this review, we will focus on novel AI applications in the clinical, endoscopic and radiologic management of pancreatitis, pancreatic cystic lesions, pancreatic ductal adenocarcinoma (PDAC) and pancreatic neuro‐endocrine tumors (pNET). An overview of the included studies is displayed in Table 1.

**Table 1:** Overview of the model characteristics in the included studies

First author (year)	Purpose of the model	Type of model	Input shape	Type of validation
Andersson *et al*. (2011)^12^	Severity prediction of AP	Conventional ML	Clinical and biochemical features	Internal
Pearce *et al*. (2006)^13^	Severity prediction of AP	Conventional ML	Clinical and biochemical features	Internal
Zhu *et al*. (2015)^14^	Differentiation of autoimmune pancreatitis and CP	Conventional ML	Radiomic features (EUS)	Internal
Mashayekhi *et al*. (2020)^15^	Differentiation of functional abdominal pain, CP and recurrent AP	Conventional ML	Radiomic features (CT)	Internal
Fei *et al*. (2018)^17^	Complication prediction in AP	Conventional ML	Clinical and biochemical features	Internal
Fei *et al*. (2017)^18^	Complication prediction in AP	Conventional ML	Clinical and biochemical features	Internal
Qiu *et al*. (2019)^19^	Complication prediction in AP	Conventional ML	Clinical and biochemical features	Internal
Hong *et al*. (2013)^20^	Complication prediction in AP	Conventional ML	Clinical and biochemical features	Internal
Qiu *et al*. (2019)^21^	Complication prediction in AP	Conventional ML	Clinical and biochemical features	Internal
Mofidi *et al*. (2007)^22^	Identification of severe AP	Conventional ML	Clinical and biochemical features	Internal
Halonen *et al*. (2003)^23^	Mortality prediction in AP	Conventional ML	Clinical and biochemical features	Internal
Keogan *et al*. (2002)^24^	Outcome prediction in AP	Conventional ML	Clinical and biochemical features	Internal
Dmitriev *et al*. (2017)^27^	Classification of pancreatic cysts	Two components: 1. ML 2. DL	1. Radiomic features (CT) 2. CT images	Internal
Li *et al*. (2018)^28^	Classification of pancreatic cysts	DL	CT images	Internal
Wei *et al*. (2019)^30^	Diagnosis of serous cystic neoplasm	Conventional ML	Clinical and radiomic features (CT)	Internal
Yang *et al*. (2019)^31^	Classification of pancreatic cysts	Conventional ML	Radiomic features (CT)	Internal
Springer *et al*. (2019)^33^	Management of pancreatic cysts	Conventional ML	Clinical, imaging, genetic and biochemical features	Internal
Kurita *et al*. (2019)^34^	Differentiation of malignant and benign pancreatic cysts	DL	Clinical, imaging and biochemical features	Internal
Kuwahara *et al*. (2019)^35^	Identification of malignancy in IPMN	DL	EUS images	Internal
Corral *et al*. (2019)^36^	Classification of IPMN	DL	MR‐images	Internal
Chakraborty *et al*. (2018)^37^	Classification of IPMN	Conventional ML	Clinical and radiomic features (CT)	Internal
Zhu *et al*. (2019)^41^	Detection of PDAC	DL	CT images	Internal
Liu *et al*. (2019)^42^	Detection of PDAC	DL	CT images	External Dataset: CT scans from 100 PDAC patients
Chu *et al*. (2019)^43^	Detection of PDAC	Conventional ML	Radiomic features (CT)	Internal
Li *et al*. (2018)^44^	Detection PDAC	Conventional ML	Radiomic features (PET–CT)	Internal
Gao *et al*. (2020)^45^	Differentiation of various pancreatic lesions/diseases	DL	MR‐images	External Dataset: MR series from 56 pancreas patients
Zhang *et al*. (2010)^47^	Differentiation of PDAC and normal tissue	Conventional ML	Radiomic features (EUS)	Internal
Das *et al*. (2008)^48^	Differentiation of PDAC, CP and normal tissue	Conventional ML	Radiomic features (EUS)	Internal
Norton *et al*. (2001)^49^	Differentiation of PDAC and pancreatitis	Conventional ML	EUS images	Training phase
Zhu *et al*. (2013)^50^	Differentiation of PDAC and CP	Conventional ML	Radiomic features (EUS)	Internal
Săftoiu *et al*. (2015)^51^	Differentiation of focal pancreatic masses	Conventional ML	Radiomic features (contrast‐enhanced EUS)	Internal
Ozkan *et al*. (2015)^52^	Detection of PDAC	Conventional ML	Radiomic features (EUS)	Internal
Săftoiu *et al*. (2008)^53^	Differentiation of PDAC and CP	Conventional ML	Imaging texture feature	Internal
Săftoiu *et al*. (2012)^54^	Differentiation of focal pancreatic masses	Conventional ML	Imaging texture feature	Internal
Zhang *et al*. (2020)^58^	Survival prediction for PDAC	DL	CT images	External Dataset: CT scans from 30 PDAC patients
Hayward *et al*. (2010)^59^	Prediction of clinical performance in PDAC patients	Conventional ML	Clinical variables	Internal validation
Walczak *et al*. (2017)^60^	Survival prediction for PDAC	Conventional ML	Clinical variables	Internal validation
Kaissis *et al*. (2019)^61^	Survival and subtype prediction of PDAC	Conventional ML	Radiomic features (MRI)	External Dataset: MR‐scans from 30 PDAC patients
Kaissis *et al*. (2019)^75^	Subtype prediction of PDAC	Conventional ML	Radiomic features (MRI)	Internal
Kaissis *et al*. (2020)^62^	Subtype prediction of PDAC	Conventional ML	Radiomic features (MRI)	Internal
Qiu *et al*. (2019)^65^	Histopathological grade prediction of PDAC	Conventional ML	Radiomic features (CT)	Internal
Li *et al*. (2019)^66^	Gene expression profile prediction of PDAC	Two components: 1. ML 2. DL	1. Radiomic features (CT) 2. CT images	Internal
Luo *et al*. (2020)^69^	Histopathological grade prediction of pNET	DL	CT images	External Dataset: CT scans from 19 pNET patients
Gao *et al*. (2019)^70^	Histopathological grade prediction of pNET	DL	MR‐images	External Dataset: MR‐scans from 10 pNET patients

AP, acute pancreatitis; CP, chronic pancreatitis; CT, computed tomography; DL, deep learning; EUS, endoscopic ultrasound; IPMN, intraductal papillary mucinous neoplasm; ML, machine learning; MR, magnetic resonance; MRI, magnetic resonance imaging; PDAC, pancreatic ductal adenocarcinoma; PET–CT, positron emission tomography – computed tomography; pNET, pancreatic neuroendocrine tumor.

## PANCREATITIS

The accuracy of models that are used in clinical practice to predict the clinical course of acute pancreatitis (AP), such as the acute physiology and chronic health evaluation II score (APACHE‐II score), remain modest. Many studies have investigated the added value of ML models in predicting the clinical course of AP.

### Detection

Two studies compared the accuracy of ML models to the APACHE‐II score in predicting the severity of AP with the use of clinical and laboratory findings.^12^, ^13^ The models reached a significantly higher area under the receiver operating curve (AUC) (0.92 and 0.82) than the APACHE‐II score (0.63 and 0.74). Zhu *et al*.^14^ established two algorithms to improve the ability to discriminate chronic pancreatitis (CP) from autoimmune pancreatitis during endoscopic ultrasound (EUS). One of those algorithms yielded an accuracy, sensitivity and specificity for diagnosing autoimmune pancreatitis of 89.3%, 84.1% and 92.5%, respectively.

A recently published paper investigated the *radiomic* CT features from patients with recurrent AP, CP and functional abdominal pain after the painful episode had disappeared.^15^ *Radiomics* is the process of extracting “hidden” quantitative imaging features from radiology images, with the purpose of providing more detailed information about areas of interest.^16^ In total, radiomics of 56 CT series were extracted and used to train a ML model which predicted the correct diagnosis in 82.1%. The positive predictive value (PPV) for functional abdominal pain was 100%, indicating that none of the cases with recurrent AP or CP were misclassified as functional complaints.

### Prediction of disease severity

Several studies report ANNs that predict complications and mortality in patients with AP with high accuracy, ranging from 83.0% to 97.5%.^17^, ^18^, ^19^, ^20^, ^21^, ^22^, ^23^ Three studies aimed to predict complications by using an ANN and compared it to logistic regression (LR) modeling. The results showed that the ANN significantly outperformed the LR modeling in predicting the occurrence of several complications during the course of the disease in all three studies.^17^, ^18^, ^19^ Two studies reported ANNs that predict multi‐organ failure (MOF) in AP patients based on clinical and laboratory findings. The first ANN was trained in 263 patients and reached an accuracy comparable to LR model, SVM, and the APACHE‐II score (0.81–0.84).^21^ Interestingly, the second ANN was trained on prospectively collected data of 312 patients and reached a significantly higher AUC (0.96) than that of LR model (0.88) and the APACHE‐II score (0.83).^20^

The use of ML models in predicting the severity of AP was investigated by two studies using both clinical and laboratory variables. After the first algorithm was trained on a dataset of 664 patients, it showed a significantly higher accuracy in severity, MOF and mortality prediction than the APACHE‐II or the Glasgow Severity (GS) scoring system.^22^ In contrast, the second algorithm was trained on a dataset of 234 patients using 16 variables. Validation of the algorithm showed no differences in accuracy between the LR model, the ANN model and the APACHE‐II score.^23^ Lastly, Keogan *et al*. explored the ability of a novel ANN to predict severe illness in patients admitted with AP. Manually derived CT features, clinical and laboratory findings were used to train the ANN. The model outperformed the conventional scoring systems in predicting whether or not a subject would exceed the mean length of stay and outperformed the conventional scoring systems.^24^

The above‐mentioned studies show that AI‐based applications might improve the prediction of disease severity, complications and mortality in patients with AP. However, some studies show conflicting results and most algorithms have not yet been validated on an external dataset.

## CYSTIC LESIONS OF THE PANCREAS

The rapid improvement and broad utilization of imaging has resulted in an increased detection of pancreatic cystic neoplasms (PCN). The management of PCN is challenging, since both the classification as the assessment of the risk of malignancy are currently suboptimal.^25^, ^26^

### Differentiation of pancreatic cystic lesions

Two studies developed algorithms to discriminate between four types of PCN on CT: intraductal papillary mucinous neoplasm (IPMN), mucinous cystic neoplasm (MCN), serous cystic neoplasm (SCN) and solid papillary neoplasm (SPN).^27^, ^28^ The first study combined demographic variables with manually selected and CNN‐based imaging features. The results showed that this model could differentiate between the types of PCN with an accuracy of 84%.^27^ These results are promising, considering the diagnostic accuracy of experienced abdominal radiologists is not higher than 70%.^29^ However, their model required manual selection of demographic and imaging features, and precise segmentation of the lesion beforehand. Important contextual information can be missed using only the lesion itself for classification. Therefore, Li *et al*. aimed to develop a CNN model to classify PCN on whole pancreas CT images. Additionally, saliency maps were generated to highlight the important pixels within the image and to visualize the critical areas that contributed to the classification output. The DL model achieved an accuracy of 73%, while the accuracy of the radiologists in this cohort was 48%.^28^ Surprisingly, the saliency maps showed that critical information was derived not only from the region around the PCN, but also from the boundaries of the pancreas, indicating that the shape of the pancreas border contributes to the eventual decision. Wei *et al*. developed a ML‐based model to differentiate between SCNs and non‐SCNs based on radiomic features from preoperative CT images.^30^ In the validation cohort, the model achieved an AUC of 0.84 and outperformed clinicians and guideline‐based features. Yang *et al*. published a preliminary study on a ML model that distinguishes SCN from MCN on CT, reporting a diagnostic accuracy of 83%.^31^

### Predicting the risk of malignancy

Even if best clinical practice according to international guidelines is applied, the differentiation between (pre)malignant and benign pancreatic cystic lesions remains challenging.^32^ Two papers showed that the use of DL models might be a helpful tool to predict the risk of malignancy in those lesions.^33^, ^34^ An international research group developed the CompCyst, a ML‐based guidance for clinical management of cystic lesions, using clinical features, imaging characteristics and genetic and biochemical markers.^33^ This comprehensive model was trained with data from 436 patients with all types of pancreatic cysts. During prospective testing on a group of 426 patients, the CompCyst showed a significantly higher accuracy of 69% than the current standard of care (56%) in either classifying patients as requiring surgery, requiring further monitoring or as not requiring follow‐up. The DL algorithm developed by Kurita *et al*.^34^ used clinical and biochemical parameters to predict the risk of malignancy in PCN. The algorithm was validated on a single‐center retrospective data set of 85 patients and yielded a significantly higher accuracy (92.9%) for predicting malignancy than CEA or cytology alone.

Three groups developed AI models specifically predicting the risk of malignancy in IPMN. Kuwahara *et al*.^35^ developed a DL model to detect malignant transformed IPMN on EUS imaging. The algorithm was trained and validated on 3790 still EUS images, reaching an accuracy of 94.0%. It showed a significantly better accuracy than human diagnosis (56%) and conventional guidelines (40–68%). Corral *et al*. proposed a CNN for the assessment of dysplasia in IPMN on MR‐images. The model had a sensitivity and specificity of 75% and 78% for recognizing high grade dysplasia or cancer. These results were comparable to an experienced radiologist following current guidelines, but the DL model performed the task in only 1.82 seconds.^36^ Chakraborthy *et al*.^37^ developed a ML model incorporating clinical and imaging features to predict high‐ or low‐risk branch‐duct (BD)‐IPMNs and reported a sensitivity of 80% with a specificity of 59%. Especially for risk prediction in PCN, it is important to aim for a high specificity with a low false positive rate to avoid unnecessary major surgery. However, the results of the discussed models are encouraging, in particular considering the relatively disappointing accuracy with currently applied international guidelines.^38^

## PANCREATIC DUCTAL ADENOCARCINOMA

Pancreatic ductal adenocarcinoma (PDAC) has one of the poorest prognoses among all cancers.^39^ The poor survival rate is predominantly caused by its late diagnosis in advanced stages that disqualifies patients for curable resection. Subtle lesions can be missed on imaging, especially in an urgent setting or in the absence of pancreatic symptoms.^40^

### Early detection

Zhu *et al*. developed a DL based segmentation‐for‐classification model to detect and segment pancreatic cancer lesions on CT. The results were promising, with a sensitivity of 94.1% and specificity of 98.5%.^41^ Similar results were found by Liu *et al*., who developed a DL‐CNN on 338 annotated CT series of patients with various stages of PDAC.^42^ The model was able to point out the tumor lesion in only 3 seconds with an AUC of 0.96. Another study reported their results on a ML‐based model distinguishing cancerous from normal pancreatic tissue using segmented pancreas CT images.^43^ Interestingly, the model classified all PDACs as cancer and only one normal case as PDAC in 125 CT series, with an AUC of 99.9%. Comparable results were found in a ML model that was trained to identify and classify PDAC on PET–CT images of 80 cases and healthy controls, reaching a detection accuracy of 96.5%.^44^ However, these studies only included images of normal pancreases and PDAC, while, in particular, the differentiation between diverse pancreatic lesions can be challenging. In light of this, Gao *et al*.^45^ recently developed a DL‐CNN that differentiates between various pancreatic lesions on MR‐images. The model was trained with annotated MR series from 398 patients with benign and malignant confirmed pancreatic diseases. A generative adversarial network (GAN) was used to augment and balance the dataset with synthetic images. In the external validation set, the accuracy was 76.8% for the DL model as compared to 82.0% by the radiologist. Cohen’s kappa coefficient between human reader and DL model was 0.89, indicating “almost perfect agreement”.

EUS is a sensitive imaging modality to discriminate between PDAC and benign diseases of the pancreas, although – especially in the presence of chronic pancreatitis – the differentiation remains difficult.^46^ The added value of AI to discriminate PDAC from benign diseases during EUS has been investigated in a considerable amount of studies.^47^, ^48^, ^49^, ^50^, ^51^, ^52^ Three study groups developed a ML model that differentiated normal pancreatic tissue from PDAC on EUS imaging with an accuracy of >93%.^47^, ^48^, ^52^ Interestingly, one study reported an increased accuracy of their algorithm when patient groups were divided by age.^52^ In distinguishing PDAC from CP on EUS images, two research groups developed algorithms that accurately predicted PDAC in >80% of cases, similar to the blinded interpretation of an experienced endosonographist.^49^, ^50^ A similar model was validated with recordings from 112 PDAC patients and 55 CP patients.^51^ Compared to the sensitivity and specificity of EUS‐FNA (84.8% and 100%) and contrast‐enhancing EUS (87.5% and 92.7%), the algorithm reached a sensitivity of 94.6% and specificity of 94.4% in discriminating PDAC from CP.

Endoscopic ultrasound‐guided elastography is gaining interest as a technique that can provide additional information about pancreatic focal lesions. Interpretation of real‐time EUS elastography results by an ANN was investigated in a multicenter prospective manner.^53^ The ANN – that was trained in discriminating benign from malignant lesions – yielded an accuracy of 95%. The same group performed another multicenter prospective study in 258 patients with CP or PDAC in which the algorithm yielded a significantly higher sensitivity (87.6%) and specificity (82.9%) than standard analysis by two experienced endoscopists (sensitivity 80.0%, specificity 50.0%).^54^

### Survival predictions

Traditional survival analysis tools assume a linear relationship between independent features and outcome, with respect to time.^55^ However, especially in diseases with a poor prognosis like pancreatic cancer, this linear assumption oversimplifies the association. Recent advances in ANN made it possible to model non‐linear and complex relationships between prognostic features and the risk of a certain outcome for a specific individual.^56^, ^57^ Zhang *et al*.^58^ created a CNN architecture to extract disease‐specific CT imaging features associated with survival patterns in PDAC. Interestingly, the model used annotated CT images and survival data from 422 non‐small cell lung cancer patients as pre‐training dataset and images from 68 PDAC patients as fine‐tuning dataset. Results showed that the CNN model outperformed the traditional model in predicting the survival of participants.

Two studies investigated the accuracy of ML in survival prediction using clinical variables.^59^, ^60^ The first study used clinical variables from 91 PDAC patients to develop several models that predict survival rates.^59^ The model achieved a significantly better performance (accuracy of 0.60) in predicting survival than the LR model (accuracy of 0.42). Another paper reported an algorithm that predicts 7‐month survival in patients with PDAC based on prospectively acquired clinical data from 219 patients.^60^ The algorithm yielded a sensitivity of 91% in predicting 7‐month survival, although specificity only reached 38%.

### Phenotyping

A German research group developed multiple ML‐algorithms to predict survival rates and molecular subtypes of PDAC from MR and CT images.^61^, ^62^ ML analysis of extracted radiomic features may predict molecular subtypes of PDAC, which is relevant for targeted treatment strategies and expected survival. Currently, molecular subtypes are assessed in a sub‐section of the sampled tumor and are therefore likely under‐representing the heterogeneity of subtypes within a tumor.^63^, ^64^ The benefit of radiomic analysis is that the whole‐tumor can be assessed before treatment and that the results can guide treatment strategy. Another recent study reported the performance of a ML‐based CT texture analysis for preoperative prediction of differentiation grades in PDAC.^65^ The model accurately predicted high grade PDAC in 86%. In addition, Li and colleagues demonstrated a significant correlation between textural features on CT, extracted by a CNN, and expression of oncogenes C‐MYC and HMGA2, which play a role in progression, dedifferentiation and metastasis of cancer cells.^66^

Recent innovations in the field of AI and the management of PDAC may further optimize patient survival by early identification, risk assessment and patient‐specific tumor classification. Establishing personalized medicine through ML may be a valuable asset in tailoring future treatment strategies.

## PANCREATIC NEUROENDOCRINE TUMOR (pNET)

Pancreatic neuroendocrine tumor (pNET) is a rare disease with an incidence of <1 per 100,000 individuals.^67^ The management and prognosis of pNET are for the greater part guided by the pathological differentiation grade, which requires biopsy or surgical resection.^68^ Luo *et al*. aimed to develop a non‐invasive DL model that predicts the pathological grading of pNET preoperatively from CT‐imaging. In an external validation set, the DL model accurately distinguished grade 1/2 from grade 3 pNETs in 82.1% of cases.^69^ Another study by Gao *et al*. trained a DL model that graded pNET using MR‐images. In the test‐set, the model reached an accuracy of 81.1% with an AUC of 0.89.^70^

## SUMMARY

In this review, we showed that AI applications for pancreatic diseases are rapidly evolving. Recent studies demonstrate promising results for both conventional ML technologies, such as DL models, that are able to facilitate clinical prediction and decision making, as well as interpretation of radiological imaging and guidance of endoscopic procedures. Although big steps have been taken in recent years, it is important to address the hurdles that still need to be overcome before these technologies can be implemented into our clinical routine.

To start, several studies in this review trained and validated their algorithm on relatively small, internally derived datasets. This implicates that the training data is rather homogeneous and therefore the models may not generalize well from training data to unseen data and might be overfitted, especially in DL models. Future efforts should demonstrate the robustness of these models in large, externally derived datasets from multiple centers. Secondly, the majority of the studies investigated algorithms that discriminate between limited possible outcomes (e.g. PDAC and CP). However, before clinical implementation, it is essential that these models are trained on more outcomes, representing *real world* outcomes. Furthermore, DL models can handle high data complexity, yet are limited in demonstrating the reasoning behind their prediction. Particularly for health care utilization, it is crucial to build trust in these models and being able to understand their prediction, not at least for regulatory purposes.^71^ Although considerable efforts have been made regarding *explainable* DL, the problem is still not solved at large.^72^

### Future perspectives

Medical imaging has developed and improved rapidly in recent years and contains far more visual information than the human eye can process. The assessment of images by humans are prone to perceptual and cognitive errors and are subject to inter‐ and intra‐observer variability.^73^ A similar expansion of captured digital information can be seen in electronic health records and social media, both offering incredible big data resources. In all likelihood, future AI technologies will anticipate these resources, e.g. identifying subjects with an increased risk for PDAC or detecting subtle lesions on medical images.^74^

In conclusion, ML methods are emerging and contributing to precision medicine in the management of pancreatic diseases. Despite the expanding knowledge and experience, several limitations need to be addressed before implementation in clinical practice. Instead of considering AI models as a substitute for human intelligence, emphasis should be made on the fact that these methods will aid in avoiding tedious tasks and inconsistency in diagnosis due to varying clinical experience and expertise.

## CONFLICT OF INTEREST

Author M.B.W. was supported by grants or donations from Fujifilm, Boston Scientific, Olympus, Medtronic, Ninepoint Medical and Cosmo/Aries Pharmaceuticals, author J.E.v.H. was supported by grants from Cook medical. Author M.B.W. owns stock of Virgo Inc, and is consulting for Virgo Inc, Cosmo/Aries Pharmaceuticals, Anx Robotica (2019), Covidien and GI Supply. On behalf of Mayo Clinic, M.B.W. is consulting for GI Supply (2018), Endokey, Endostart, Boston Scientific and Microtek and received general payments/minor food and beverage from Synergy Pharmaceuticals, Boston Scientific and Cook Medical. Author J.E.v.H. received consultancy fees from Boston Scientific, Medtronics and Cook Medical. Authors M.G. and S.A.H. declare no Conflict of Interests for this article.

## FUNDING INFORMATION

The funding source had no role in the design, practice or analysis of this study.

## Supporting information


**Table S1** Systematic literature search.
