# Supplementary material for: Artificial intelligence for the management of pancreatic diseases
Source: Dig Endosc. 2020 Dec 5;33(2):231–41. doi: 10.1111/den.13875 (PMC7898901; doi:10.1111/den.13875)
Supplement: Supplementary file 1 — Table S1 Systematic literature search. [file DEN-33-231-s001.docx]

| **Database** | Search | Results |
| --- | --- | --- |
| PubMed (01-06-2020) | ("Pancreatic Diseases"[Mesh] OR "Biliary Tract Diseases"[Mesh] OR pancrea*[tiab] OR biliary tract[tiab] OR biliary system[tiab] OR cholangio*[tiab] OR bile*[tiab] OR gallbladder[tiab]) AND ("Artificial Intelligence"[MAJR] OR "Diagnosis, Computer-Assisted"[MAJR] OR machine learning*[tiab] OR artificial intelligen*[tiab] OR deep learning[tiab] OR neural network*[tiab] OR computer-assisted[tiab] OR computer-aided[tiab]) AND ("diagnosis" [Subheading] OR "diagnostic imaging" [Subheading] OR imaging[tiab] OR endoscop*[tiab] OR endoscopy[MeSH Terms])  Filters: English, from 2000 - 2020 Sort by: Publication Date | 859 |

*Table S1: Systematic literature search*
